# Antioxidant Enzyme Activities and Lipid Oxidation in Rape (*Brassica campestris* L.) Bee Pollen Added to Salami during Processing

**DOI:** 10.3390/molecules21111439

**Published:** 2016-10-28

**Authors:** Yawei Zhang, Fengtian Yang, Muneer Ahmed Jamali, Zengqi Peng

**Affiliations:** 1 College of Food Science and Technology, National Center of Meat Quality and Safety Control, Nanjing Agricultural University, Nanjing 210095, China; zhangyawei@njau.edu.cn (Y.Z.); 2014808124@njau.edu.cn (F.Y.); majamali65@yahoo.com (M.A.J.); 2 Synergetic Innovation Center of Food Safety and Nutrition, Nanjing 210095, China

**Keywords:** rape bee pollen (RBP), salami, antioxidant enzyme, lipid oxidation

## Abstract

The present research investigated the antioxidant effect of rape (*Brassica campestris* L.) bee pollen (RBP) on salami during processing. Eight flavonoids in RBP ethanol extract were quantified by high-performance liquid chromatography-mass spectrometry (HPLC-MS) analysis, and quercetin, rutin, and kaempferol were the major bioactive compounds. The RBP ethanol extract exhibited higher total antioxidant capacity than 6-hydroxy-2,5,7,8-tertramethylchromancarboxylic acid (trolox) at the same concentration. The salami with 0.05% RBP extract had higher catalase (CAT), superoxide dismutase (SOD), and glutathione peroxidase (GSH-Px) activities than that of the control throughout the processing time (*p* < 0.05). Significant decreases in peroxide value (POV) and thiobarbituric acid-reactive substances (TBARS) were obtained in the final salami product with 0.05% RBP ethanol extract or 1% RBP (*p* < 0.05). These results suggested that RBP could improve oxidative stability and had a good potential as a natural antioxidant for retarding lipid oxidation.

## 1. Introduction

Salami is susceptible to lipid oxidation [1,2,3] because of its 30% fat content [4]. Usually, the best strategy to minimize lipid oxidation is the addition of antioxidants during processing [5]. The search for natural antioxidants has increased considerably in recent decades, because of toxicological concerns and health issues of several synthetic antioxidants [6,7]. Many natural antioxidants were found to possess lipid-protective effects in meat products. Qi et al. reported that the addition of lychee seed extract inhibited adipogenesis and retarded lipid oxidation in meat paste during storage [8]. Rosemary extract was found to be effective in inhibiting the amount of malondialdehyde (MDA) content in pork sausage [9] and goat meat sausage [10]. In salami sausage, Campagnol et al. reported that the addition of 0.05% *Achyrocline satureioides* extract caused a significant decrease of 35.7% in TBARS values compared to the control after 75 days of storage and retarded the lipid oxidation during the storage period [11]. Though there are a large number of compounds that have been proposed to possess antioxidant activity, only a few can be used in food products because the use of antioxidants in food products is controlled by the regulatory laws of a country or international standards [12].

Bee pollen, an apicultural product, has been used as a “perfect health food” and as a dietary supplement for humans for many centuries [13,14]. It possesses high nutritional value and physiological properties in human nutrition [15] and has attracted considerable attention in recent years. The ethanolic extract from sweet maize pollen was determined to have abundant flavonoids, and the fresh pollen has 104.38 mmol trolox eq/kg antioxidant capacity in vitro [13]. Rape bee pollen (RBP), commonly used as a nutritional food and a traditional medicine [16], is rich in flavonoids [17]. It was found that the aqueous extract from RBP decreased the MDA levels and increased the GSH-Px activity in the liver of rats treated with propoxur [18] or carbaryl [19]. However, little information is available concerning the antioxidant effect of RBP on meat products during processing, particularly salami.

Meat has endogenous antioxidants, including antioxidant enzymes, such as superoxide dismutase (SOD), catalase (CAT), and glutathione peroxidase (GSH-Px) [20]. Therefore, the present study was undertaken to evaluate the influence of RBP on the endogenous antioxidant enzymatic activities and lipid oxidation in salami during processing. Additionally, identification and quantification of flavonoid compounds, total flavonoid, and the total antioxidant capacity of RBP extract were also evaluated, in order to provide information about the potential use of rape bee pollen as a natural food-grade antioxidant in salami.

## 2. Results and Discussion

### 2.1. Identification and Quantification of Flavonoid Compounds

**Figure 1:**
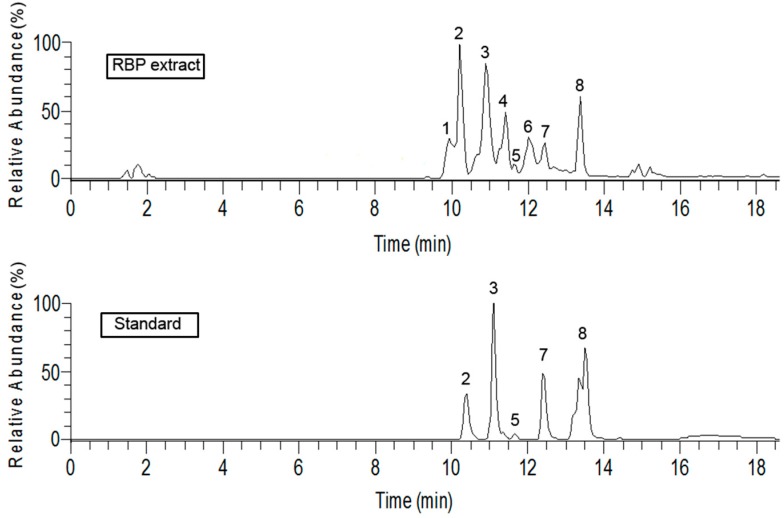
Total ion chromatograms of RBP extract and standard substances. Note: standard 2 = rutin, standard 3 = quercitrin, standard 5 = quercetin, standard 7 = kaempferol, and standard 8 = isorhamnetin.

The total ion chromatograms of the flavonoids in the RBP extract and of the standard substances are shown in Figure 1. The five components and the standard had the same retention time, molecular ion, and MS^2^ spectrum under the same analytical conditions. The three peaks resulting from unknown substances in the chromatograms were identified by MS/MS. Peak 1 showed a molecular ion [M − H]^−^ at 463.09, MS^2^: 301.03, 463.08, for C_21_H_19_O_12_, with an error of 2.889 ppm. Peak 4 has a molecular ion [M − H]^−^ at 447.09, MS^2^: 447.09, 285.04 for C_21_H_19_O_11_, with an error of 2.435ppm. The molecular ion of peak 6 was [M − H]^−^ 271.0603, MS^2^: 151.01, 177.02, 119.05, for C_15_H_11_O_5_, with an error of 3.618 ppm. Thus, peaks 1, 4, and 6 identified as quercetin 3-*O*-glucoside, kaempferol 3-*O*-glucoside [21], and naringenin [22,23], respectively.

The flavonoids in the RBP extract are quercetin 3-*O*-glucoside, rutin, quercitrin, kaempferol 3-*O*-glucoside, quercetin, naringenin, kaempferol, and isorhamnetin. Kaempferol (145.15 mg/g), quercetin (38.68 mg/g), and rutin (35.52 mg/g) are the major bioactive compounds found in the RBP extract (Table 1), whereas rutin and quercetin are the dominant component of flavonoids in rape bee pollen [24] and maize pollen [13], respectively. This slight difference was observed in our study might be due to the environmental conditions and the plant species from which the pollen was gathered [25,26].

### 2.2. Total Antioxidant Capacity of RBP Extract

**Figure 2:**
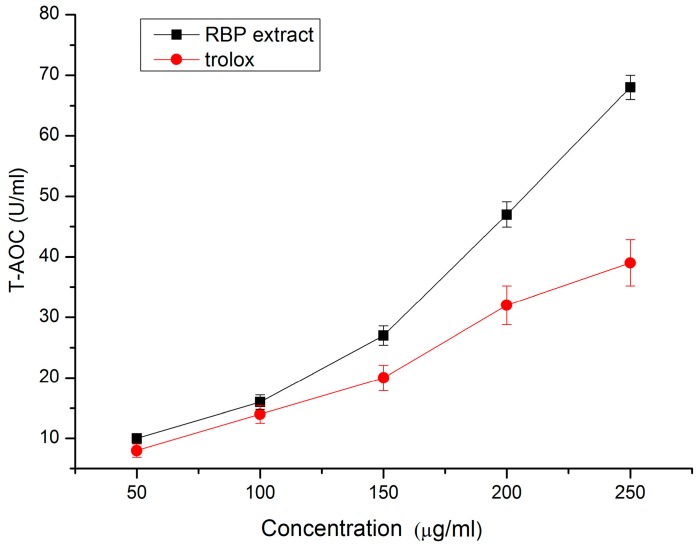
Total antioxidant capacity of RBP extract and trolox.

At concentrations from 50–250 μg/mL, the total antioxidant capacity (T-AOC) of the RBP extract ranged from 2.1–68.1, whereas the trolox T-AOC ranged from 1.2–39.7 (Figure 2). At concentrations of 200 μg/mL and 250 μg/mL, the T-AOC of the RBP extract was 46.9% and 74.4% more than that of trolox, respectively (*p* < 0.05), suggesting that the RBP extract possessed higher total antioxidant capacity than trolox. This result was in accordance with the study in *Cistus ladaniferus* pollen extract, rich in flavonoids, showing a high protective effect on lipid oxidation [24].

### 2.3. Effect of RBP Extract on the Endogenous Antioxidant Enzymatic Activities in Salami during Processing

The total flavonoid content of the RBP extract was 604 mg rutin equivalent/g. Considering the total flavonoid contents of the RBP extracts (604 mg/g), 1 g RBP extract was equivalent to 0.60 g rutin, quercetin, or kaempferol, respectively, i.e., 0.05% RBP extract used in salami was equivalent to 0.03% rutin, quercetin, or kaempferol.

#### 2.3.1. GSH-Px Activity

**Figure 3:**
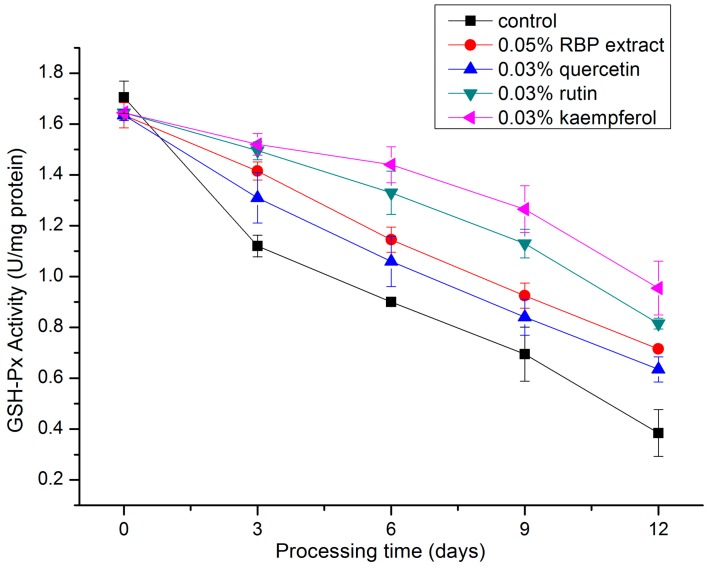
Changes in GSH-Px activity during the processing of salami with different treatments.

GSH-Px is known as an important cellular scavenger of hydroxyl radicals [27] and has been observed in many dry-cured meat products [28,29]. It catalyzed the reduction of hydroperoxides to non-toxic products and terminated the chain reaction of lipid peroxidation by removing lipid hydroperoxides [30]. As shown in Figure 3, the GSH-Px activity decreases in salami during processing in all formulations. At the end of processing (the twelfth day), the GSH-Px activity of each treatment were 1.86-fold (0.05% RBP extract), 1.65-fold (0.03% quercetin), 2.27-fold (0.03% rutin), and 2.48-fold (0.03% kaempferol) compared to that of the control. The order of the protective GSH-Px efficiency of the RBP extract and the flavonoids was kaempferol > rutin > RBP extract > quercetin. These findings are supported by the study of Cheng et al., who found that *S. chinensis* bee pollen extract decreased the extent of MDA formation and elevated the GSH-Px activity in mouse liver, indicating that the *S. chinensis* bee pollen extract had strong antioxidant activities [31]. The effect of kaempferol on oxidative stress in the mouse model of Parkinson’s disease described by Li and Pu showed that kaempferol increased the GSH-Px activity and retarded oxidative stress [32].

#### 2.3.2. CAT Activity

**Figure 4:**
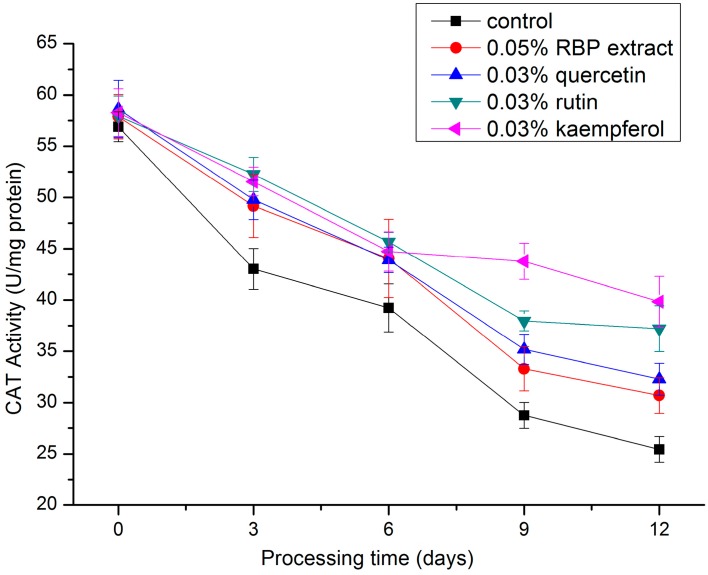
Changes in CAT activity during the processing of salami with different treatments.

A decreasing trend with increasing processing days was observed for CAT activity in the salami during processing (Figure 4), and the CAT activities of the treatment groups were significantly higher (*p* < 0.05) than that of the control group. On the twelfth day, the CAT activities of the treatment groups were 1.21-fold (0.05% RBP extract), 1.27-fold (0.03% quercetin), 1.46-fold (0.03% rutin), and 1.56-fold (0.03% kaempferol) higher than that of the control group. This result illustrates that RBP extract and these flavonoids increased the CAT activity in salami during processing. The protective effect is consistent with a previous study [33], indicating that the CAT activity in the liver of mice fed with *C. incanus* L. bee pollen was significantly increased compared to the control. Additionally, increased CAT activity was found in the pancreatic tissue of rats [34] and in the liver of mice [35] treated with quercetin. Hu et al. showed that the CAT activity in breast muscles of chicken supplemented with broccoli stem and leaf was 17.95% greater than that of the control group due to the synergistic effects of kaempferol, quercetin, and other flavonoids [36].

#### 2.3.3. SOD Activity

**Figure 5:**
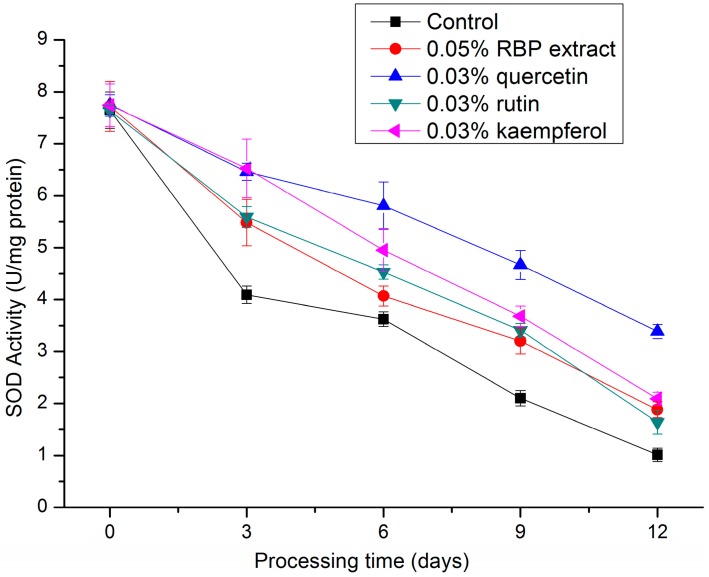
Changes in SOD activity during the processing of salami with different treatments.

SOD is an effective defense enzyme that catalyzes the dismutation of superoxide anions into hydrogen peroxide (H_2_O_2_) [37], i.e., catalyzing the dismutation of 2O_2_^−^ + 2H**^+^** into H_2_O_2_ + O_2_. It accelerated the spontaneous reaction of dismutation of the superoxide radical anion by a cyclic oxidation-reduction mechanism of an active site metal ion. For mammalian tissues, CuZnSOD constituted 85%–90% of total SOD activity and MnSOD usually accounted for 10%–15% [38]. As shown in Figure 5, activity of SOD in the treatment groups were significantly higher (*p* < 0.05) than that of the control. At the end of processing, the SOD activity in 0.03% quercetin, 0.03% kaempferol, 0.05% RBP extract. and 0.03% rutin treatment was 3.36-fold, 2.07-fold, 1.86-fold, and 1.62-fold higher than that of the control group, respectively, showing that the RBP extracts and these flavonoids increased the SOD activity in salami during processing. Cheng et al. found that the hepatic SOD activity in mice treated with 40 mg/kg of *S. chinensis* pollen ethanol extract was significantly increased by 9.29% compared to a control group and noted that ethanolic extract of *S. chinensis* pollen could effectively protect the lipid oxidation, due to the high content of flavonoids [31].

As seen in Table 2, the average antioxidant enzyme activity was statistically calculated according to treatments and salami processing days, respectively. It was shown that treatments, processing days, and the interaction between treatments and processing days had a significant effect (*p* < 0.001) on the activity of SOD, CAT, and GSH-Px. As expected, the activity of antioxidant enzymes in salami was decreased with the processing days, while the RBP extract and flavonoids added to salami caused an increase in the SOD, CAT, and GSH-Px activity. It was suggested that RBP extract and flavonoids delayed the decreases of antioxidant enzyme activity in the salami.

### 2.4. Effect of RBP or RBP Extract on Lipid Oxidation in Salami

Due to the content of flavonoids in the RBP (30.4 mg rutin equivalent/g), considering the total flavonoids content of the RBP extract (604 mg rutin equivalent/g), 1% RBP is equivalent to 0.05% RBP extract. The results of the effect of 1% RBP or 0.05% RBP extract on lipid oxidation in salami are shown in Table 3. The peroxide values (POV) are significantly increased within nine processing days, and at the end of processing (day 12), an obvious decrease in POV was obtained in all of the treatments (*p* < 0.05). The reason for the phenomenon is probably that POV measures the amount of hydroperoxides, formed as primary oxidation products, at the initial stage of oxidation and these peroxides are unstable over time [39]. During processing, no significant difference in POV was found between the 0.05% RBP extract and the 1% RBP treatments (*p* > 0.05). Whereas, in the final products, the POV in the 0.05% RBP extract treatment and in the 1% RBP treatment was 23.24% and 21.76% lower than that in the control group, respectively (*p* < 0.05).

TBARS measures malonaldehyde, a secondary product formed by oxidation of fatty acids [40]. At day 6, MDA content was significantly lower (*p* < 0.05) in 1% RBP or 0.05% RBP extract compared to the control. At the end of processing, the TBARS in the 0.05% RBP extract and 1% RBP treatment was 34.09% and 22.7% lower than that in control, respectively (*p* < 0.05). No significant difference was found in TBARS between the 0.05% RBP extract and the 1% RBP treatments throughout processing (*p* > 0.05).

Table 4 showed that treatments, processing days, and the interaction between treatments and processing days had a significant effect (*p* < 0.01) on the POV and TBARS. The POV and TBARS of salami were both increased with processing days, while 0.05% RBP extract or the 1% RBP caused a decrease in POV and TBARS. Furthermore, there was no significant difference in POV and TBARS between the 0.05% RBP extract and the 1% RBP treatments, indicating that RBP or RBP extract had a positive effect on inhibition of lipid oxidation in salami.

The free radical-scavenging potential of flavonoids is considered to be related to the location and number of free −OH groups on the flavonoid skeleton [41] or on the aromatic ring [42]. Quercetin, rutin, and kaempferol are the major bioactive compounds in the RBP extract, and have different hydroxylation patterns, i.e., quercetin (3,5,7,3′,4′ −OH), rutin (3-rut, 5,7,3′,4′ −OH), and kaempferol (3,5,7,4′ −OH). Silva, et al. found that the presence of 3′, 4′-OH in the B-ring was a determinant for high antioxidant capacity in flavonoids, such as quercetin, kaempferol, and rutin, which shows high antioxidant potencies against lipid oxidation [42]. Furthermore, the total flavonoids in the RBP extract and RBP were 604 mg/g and 30.4 mg/g in the present study, respectively. The antioxidant effect also might be influenced by synergistic or antagonistic interactions between flavonoids [43].

## 3. Materials and Methods

### 3.1. Preparation of RBP Extract

Rape bee pollen was collected by beekeepers at an apiary in Nanjing, Jiangsu province, China, in March 2015. The bee pollen was dried in an oven (DHG-9240A, SANFA Scientific Instruments Co., Ltd., Shanghai, China) at 50 °C until a constant weight was achieved. Samples were ground for 60 s in a disintegrator (HY-04B, Tianyuan Machinery Co., Ltd., Beijing, China) and later passed through a 60-mesh sieve. The RBP powder was soaked with 80% (*v*/*v*) ethanol in the ratio of solvent to material 50 mL/g. Thereafter, ultrasound was used at 40 kHz for 30 min at 80 °C, and filtered through 0.45 μm microporous membrane (Shanghai WanziShiye Co., Ltd., Shanghai, China). The crude extracts were purified using a D101 macroporous adsorption resin according to the method of Yang et al. [44]. The RBP extract was then freeze-dried, vacuum-packaged, and stored at 4 °C until analyzed.

### 3.2. Total Flavonoid Content

The total flavonoid content of the RBP extract was measured by the method of Zhishen et al. with minor modifications [45]. The RBP extract was dissolved into 60% ethanol (*v*/*v*) and 0.4 mL of 5% NaNO_2_ (*w*/*v*) was added. After 5 min, 0.4 mL of 10% AlCl_3_ (*w*/*v*) was added to the solution. After 6 min, 4 mL of 4% NaOH was added, and the mixture was mixed well. The blank was prepared the same as reaction mixture for the total flavonoid without the RBP extract. The absorbance of the mixture was determined at 510 nm. The total flavonoid content of the RBP extract was determined using a standard curve with rutin (*y* = 1.0049*x* + 0.0014, R^2^ = 0.9966) and expressed as mg rutin equivalent/g.

### 3.3. Identification and Quantification of Flavonoid Compounds

The determination of the flavonoids in the RBP extract was performed according to the method of Li et al. with some modifications [46], using a UHPLC-LTQ-Orbitrap system (Dionex, Thermo Scientific, Sunnyvale, CA, USA). Separations were conducted with an ACQUITY UPLCTM BEH C18 (100 mm × 2.10 mm, 1.7 μm particle sizes, Waters, Milford, MA, USA) using gradient elution with a binary mobile phase (phase A: 0.1% formic acid in 5% acetonitrile, phase B: 100% acetonitrile) at 30 °C. The gradient elution program was as follows: 0 min, 100:0 (A:B); 10 min, 75:25; 21 min, 75:25; 22 min, 0:100; and 30 min, 0:100. The injection volume was 10 μL and the flow rate was 0.2 mL/min. The mass spectrometer was operated with a scan range of 150 to 1000 *m*/*z*. Nitrogen was used as a nebulizing gas with a flow rate of 810 L/h, an ionization temperature was180 °C, a spray voltage of 3500 V, and the collision gas was argon.

### 3.4. Total Antioxidant Capacity of RBP Extract

The total antioxidant capacity (T-AOC) was determined by using commercial kits (Nanjing Jiancheng Bioengineering Institute, Nanjing, China). In the reaction mixture, ferric ion was reduced by antioxidant reducing agents, and a blue complex, Fe^2+^-TPTZ (2,4,6-tri(2-pyridyl)-s-triazine), was produced. Trolox was used as control. The absorbance was measured at 520 nm. One unit (U) of T-AOC is defined as the amount that increased the absorbance by 0.01 at 37 °C. The analysis was performed in triplicate.

### 3.5. Preparation of Salami Samples

Fresh pork shoulder (70%) and back fat (25%) were minced in a rotary screw mincer (TS-8. FTSM101E, FAMA, Milan, Italy). Next, the other ingredients (sodium ascorbate, glucose, nitrite salt, starter culture, and sodium chloride) were mixed into the batter. For antioxidant enzyme activity analysis, RBP extract, rutin, quercetin, and kaempferol were added according to the following formulation: (1) control (no antioxidant added); (2) 0.05% RBP extract; (3) 0.03% rutin; (4) 0.03% quercetin; and (5) 0.03% kaempferol. For salami lipid oxidation analysis, three formulations were considered: (1) control (no antioxidant added); (2) 1% RBP; and (3) 0.05% RBP extract. The mixture obtained was stuffed into casings, and the fresh salami underwent a three-day fermentation process at 25 ± 1 °C and 95% relative humidity (RH). The salami samples were subsequently dried for another nine days. During the drying process, the temperature was 20 ± 2 °C and the RH was reduced to 75%.

The salami sausages from each formulation at five processing points (day 0, day 3, day 6, day 9, and day 12) were collected for analysis. Collected samples were vacuum-packaged and stored at −40 °C until analysis. All analyses were performed in triplicate.

### 3.6. Measurement of Endogenous Antioxidant Enzyme Activities of Salami

Samples for enzyme activity analysis were prepared according to the method of Hernández et al. with slight modifications [20]. Five grams of minced sample was homogenized in 25 mL phosphate buffer (0.05 M, pH 7) using a Polytron (60 s, 12,000 rpm) homogenizer IKA T18 basic (Ika, Staufen, Germany). During homogenization, the tubes were kept in ice to avoid heating. The homogenized sample was centrifuged at 7000× *g* for 20 min at 4 °C. The supernatant was used to determine the GSH-Px, CAT, and SOD activities.

#### 3.6.1. GSH-Px Activity

The GSH-Px activity was analyzed according to the method of Lawrence and Burk [47]. The assay mixture consisted of 1.0 mL of 75 mM phosphate buffer (pH 7.0), 10 μL of 150 mM reduced glutathione, 10 μL of 46 U/mL glutathione reductase, 30 μL of 25 mM ethylenediaminetetraacetic acid (EDTA), 30 μL of 5 mM nicotinamide adenine dinucleotide phosphate (NADPH), 200 μL of enzyme extract solution, and 10 μL of 20% TritonX-100. The reaction was started by the addition of 50 μL of 7.5 mM H_2_O_2_. The rate of change in absorbance was recorded at 340 nm for 3 min. The GSH-Px activity is expressed as U/mg protein, and one unit is defined as micromoles of NADPH oxidized to NADP^+^/min at 22 °C.

#### 3.6.2. CAT Activity

The CAT activity assay was based on the method described by Cakmak and Marschner [48], with slight modification. The reaction mixture contained 100 μL enzyme extract solution and 2.9 mL phosphate buffer (pH 7.0) with 10 mM H_2_O_2_. The CAT activity was determined by the decrease in the absorbance of H_2_O_2_ at 240 nm during the initial 3 min, and results were expressed as U/mg protein.

#### 3.6.3. SOD Activity

The SOD activity was determined by the method of Marklund [49]. The assay mixture consisted of 1.8 mL of 50 mM Tris-HCl buffer containing 10 mM EDTA, 0.1 mL of 6 mM pyrogallol, 100 μL enzyme solution (without enzyme as a blank). The absorbance was measured at 420 nm for 10 min, and results were expressed as U/mg protein.

### 3.7. Salami Lipid Oxidation

The lipid oxidation was evaluated by peroxide value (POV) and 2-thiobarbituric acid-reactive substances (TBARS). The POV was measured according to the method of Shanta and Decker [50]. The TBARS was determined according to the method of Salih et al. [51]. TBARS is expressed as mg of malondialdehyde (MDA) per kg of meat.

### 3.8. Statistical Analysis

Analysis of variance (ANOVA) was performed using SAS 8.2 (SAS Institute Inc., Cary, NC, USA). Significant differences (*p* < 0.05) between means were identified using Duncan’s multiple range tests.

## 4. Conclusions

An ethanolic extract of RBP contains abundant flavonoids, particularly quercetin, rutin, and kaempferol, and possesses high total antioxidant capacity. Throughout the entire processing, the addition of the RBP extract and the flavonoids delayed the decreases of the endogenous antioxidant enzyme (GSH-Px, SOD, and CAT) activities. Both the RBP extract and the RBP caused an evident decrease in the POV and TBARS of the salami. Furthermore, no significant (*p* > 0.05) difference was observed for the inhibition of lipid oxidation in salami between the RBP extract and RBP treatments, indicating that the RBP could be useful in ameliorating the oxidative damage and played an important role in the protection against lipid oxidation.

## Figures and Tables

**Table 1:** The retention time and fragment ions of each compound in RBP extract and standards.

Peak	Compounds	[M − H]^−^ (*m*/*z*)	RT (min) Sample	RT (min) Standard	Predicted Formula	Calibration Curve	ppm	Linear Range (μg/mg)	R^2^	Content (mg/g)	LOQ (ng/mL)
1	Quercetin 3-*O*-glucoside	463.0853	9.94	-	C_21_H_19_O_12_	-	−2.889	-	-	20.21 ± 0.02 ^a^	-
2	Rutin	609.1429	10.39	10.44	C_27_H_29_O_16_	*y* = 2.6985 × 10^6^*x* − 412038	−5.299	0.1–100	0.99	35.52 ± 0.01	100
3	Quercitrin	447.0906	10.99	11.10	C_21_H_20_O_11_	*y* = 3.9898 × 10^6^*x* − 3144208	−5.982	0.1–100	0.99	21.25 ± 0.01	100
4	Kaempferol 3-*O*-glucoside	447.0909	11.40	-	C_21_H_19_O_11_	-	−2.435	-	-	19.13 ± 0.02 ^a^	-
5	Quercetin	301.0339	11.71	11.73	C_15_H_9_O_7_	*y* = 7.263 × 10^6^*x* − 3671831	−5.002	0.1–100	0.99	38.68 ± 0.01	100
6	Naringenin	271.0603	12.09	-	C_15_H_11_O_5_	-	−3.618	-	-	26.57 ± 0.02 ^a^	-
7	Kaempferol	285.0394	12.46	12.49	C_15_H_9_O_6_	*y* = 7.640 × 10^6^*x* − 67030	−3.308	0.1–100	0.99	145.15 ± 0.04	100
8	Isorhamnetin	315.0495	13.38	13.50	C_16_H_11_O_7_	*y* = 6.1311 × 10^6^*x*− 2323606	−4.748	0.01–100	0.99	3.25 ± 0.01	10

Note: ^a^ = Internal standard method.

**Table 2:** Two-way ANOVA for different treatments, processing days, and their interaction on enzyme activity.

Effect Tested	SOD Activity	CAT Activity	GSH-Px Activity
Treatments (T)			
Control	3.70 ± 2.34d	38.67 ± 11.66d	0.96 ± 0.46e
0.05% RBP extract	4.47 ± 2.08c	43.02 ± 10.66c	1.18 ± 0.33c
0.03% Quercetin	5.62 ± 1.55a	43.96 ± 10.08b,c	1.10 ± 0.36d
0.03% Rutin	4.56 ± 2.09c	46.21 ± 8.44a,b	1.27 ± 0.31b
0.03% Kaempferol	5.00 ± 2.08b	47.63 ± 6.96a	1.37 ± 0.25a
Significance	***	***	***
Processing days (D)			
0	7.70 ± 0.10A	57.92 ± 1.95A	1.65 ± 0.04A
3	5.63 ± 0.92B	49.15 ± 3.81B	1.37 ± 0.15B
6	4.60 ± 0.78C	43.52 ± 3.01C	1.18 ± 0.20C
9	3.41 ± 0.86D	35.80 ± 5.34D	0.97 ± 0.22D
12	2.00 ± 0.81E	33.09 ± 5.47E	0.70 ± 0.20E
Significance	***	***	***
Interaction T × D	***	***	***

Note: *** = *p* < 0.001; different small letters (a–e) in the treatments and capital letters (A–E) in the processing days means significant differences.

**Table 3:** The effect of RBP on lipid oxidation during processing of salami.

Lipid Oxidation Indices	Treatments	Processing Time (Days)				
		0	3	6	9	12
POV	control	1.94 ± 0.18 ^j^	4.65 ± 0.28 ^h^	6.13 ± 0.08 ^d,e^	8.72 ± 0.41 ^a^	8.09 ± 0.13 ^b^
	0.05% RBP extract	1.99 ± 0.09 ^j^	4.23 ± 0.29 ^i^	5.76 ± 0.11 ^f^	7.21 ± 0.14 ^c^	6.21 ± 0.21 ^d^
	1% RBP	2.09 ± 0.24 ^j^	3.96 ± 0.13 ^i^	5.58 ± 0.14 ^f,g^	7.22 ± 0.19 ^c^	6.33 ± 0.11 ^d^
TBARS	control	0.23 ± 0.01 ^h^	0.32 ± 0.03 ^g^	0.57 ± 0.11 ^d,e^	0.87 ± 0.11 ^c^	1.32 ± 0.12 ^a^
	0.05% RBP extract	0.22 ± 0.01 ^h^	0.33 ± 0.02 ^g^	0.37 ± 0.04 ^g,f^	0.66 ± 0.14 ^d^	0.97 ± 0.09 ^b^
	1% RBP	0.23 ± 0.01 ^h^	0.34 ± 0.02 ^g^	0.49 ± 0.03 ^e,f^	0.71 ± 0.14 ^d^	1.02 ± 0.09 ^b^

Note: ^a–j^ Means with different superscripts letters differ significantly (*p* < 0.05).

**Table 4:** Two way ANOVA for different treatments, processing days, and their interaction on lipid oxidation.

Effect Tested	POV	TBARS
Treatment (T)		
Control	5.91 ± 2.55a	0.66 ± 0.42a
0.05% RBP extract	5.04 ± 1.89b	0.56 ± 0.30b
1% RBP	5.08 ± 1.89b	0.49 ± 0.25b
Significance	***	***
Processing days (D)		
0	2.01 ± 0.17E	0.23 ± 0.01D
3	4.28 ± 0.37D	0.33 ± 0.02D
6	5.83 ± 0.26C	0.48 ± 0.10C
9	7.72 ± 0.79A	0.75 ± 0.15B
12	6.88 ± 0.92B	1.07 ± 0.22A
Significance	***	***
Interaction T × D	***	**

Note: ** = *p* < 0.01; *** = *p* < 0.001; different small letters (a,b) in the treatments and capital letters (A–E) in the processing days means significant differences.

## References

[1] Hur S.J., Park G.B., Joo S.T. (2007). Formation of cholesterol oxidation products (COPs) in animal products. Food Control.

[2] Wójciak K.M., Dolatowski Z.J. (2012). Oxidative stability of fermented meat products. Acta Sci. Pol. Technol. Aliment..

[3] Sammet K., Duehlmeier R., Sallmann H.P., von Canstein C., von Mueffling T., Nowak B. (2006). Assessment of the antioxidative potential of dietary supplementation with α-tocopherol in low-nitrite salami-type sausages. Meat Sci..

[4] Wirth F. (1991). Reducing the fat and sodium content of meat products. What possibilities are there?. FleischWirtschaft.

[5] Kim S.J., Cho A.R., Han J. (2013). Antioxidant and antimicrobial activities of leafy green vegetable extracts and their applications to meat product preservation. Food Control.

[6] Soong Y.Y., Barlow P.J. (2004). Antioxidant activity and phenolic content of selected fruit seeds. Food Chem..

[7] Madhavi D.L., Salunkhe D.K. (1995). Toxicological aspects of food antioxidants. Food Sci. Technol..

[8] Qi S., Huang H., Huang J., Wang Q., Wei Q. (2015). Lychee (*Litchi chinensis Sonn*.) seed water extract as potential antioxidant and anti-obese natural additive in meat products. Food Control.

[9] Sebranek J.G., Sewalt V.J.H., Robbins K., Houser T.A. (2005). Comparison of a natural rosemary extract and BHA/BHT for relative antioxidant effectiveness in pork sausage. Meat Sci..

[10] Nassu R.T., Gonçalves L.A.G., da Silva M.A.A.P., Beserra F.J. (2003). Oxidative stability of fermented goat meat sausage with different levels of natural antioxidant. Meat Sci..

[11] Campagnol P.C.B., Fries L.L.M., Terra N.N., Santos B.A.D., Furtado A.S., Toneto E.R.L., Campos R.M.L.D. (2011). The influence of *Achyrocline satureioides* (“Marcela”) extract on the lipid oxidation of salami. Food Sci. Technol..

[12] Karre L., Lopez K., Getty K.J.K. (2013). Natural antioxidants in meat and poultry products. Meat Sci..

[13] Žilić S., Vančetović J., Janković M., Maksimović V. (2014). Chemical composition, bioactive compounds, antioxidant capacity and stability of floral maize (*Zea mays* L.) pollen. J. Funct. Foods.

[14] LeBlanc B.W., Davis O.K., Boue S., DeLucca A., Deeby T. (2009). Antioxidant activity of Sonoran Desert bee pollen. Food Chem..

[15] Abouda Z., Zerdani I., Kalalou I., Faid M., Ahami M.T. (2011). The antibacterial activity of Moroccan bee bread and bee-pollen (fresh and dried) against pathogenic bacteria. Res. J. Microbiol..

[16] Wagenlehner F.M., Schneider H., Ludwig M., Schnitker J., Brähler E., Weidner W. (2009). A pollen extract (Cernilton) in patients with inflammatory chronic prostatitis-chronic pelvic pain syndrome: A multicentre, randomised, prospective, double-blind, placebo-controlled phase 3 study. Eur. Urol..

[17] Lv H., Wang X., He Y., Wang H., Suo Y. (2015). Identification and quantification of flavonoid aglycones in rape bee pollen from Qinghai-Tibetan Plateau by HPLC-DAD-APCI/MS. J. Food Compos. Anal..

[18] Eraslan G., Kanbur M., Silici S., Liman B.C., Altınordulu Ş., Sarıca Z.S. (2009). Evaluation of protective effect of bee pollen against propoxur toxicity in rat. Ecotoxicol. Environ. Saf..

[19] Eraslan G., Kanbur M., Silici S. (2009). Effect of carbaryl on some biochemical changes in rats: The ameliorative effect of bee pollen. Food Chem. Toxicol..

[20] Hernández P., Zomeño L., Ariño B., Blasco A. (2004). Antioxidant, lipolytic and proteolytic enzyme activities in pork meat from different genotypes. Meat Sci..

[21] Sánchez-Rabaneda F., Jáuregui O., Casals I., Andrés-Lacueva C., Izquierdo-Pulido M., Lamuela-Raventós R.M. (2003). Liquid chromatographic/electrospray ionization tandem mass spectrometric study of the phenolic composition of cocoa (Theobroma cacao). J. Mass Spectrom..

[22] N’Dri D., Calani L., Mazzeo T., Scazzina F., Rinaldi M., Del Rio D., Brighenti F. (2010). Effects of different maturity stages on antioxidant content of Ivorian Gnagnan (*Solanum indicum* L.) berries. Molecules.

[23] Ribas-Agustí A. (2012). A validated HPLC-DAD method for routine determination of ten phenolic compounds in tomato fruits. Food Anal. Methods.

[24] Kaškonienė V., Ruočkuvienė G., Kaškonas P., Akuneca I., Maruška A. (2015). Chemometric Analysis of Bee Pollen Based on Volatile and Phenolic Compound Compositions and Antioxidant Properties. Food Anal. Methods.

[25] Arruda L., Beneduzi A., Martins A., Lisboa B., Lopes C., Bertolo F., Passaglia L.M.P., Vargas L.K. (2013). Screening of rhizobacteria isolated from maize (*Zea mays* L.) in Rio Grande do Sul State (South Brazil) and analysis of their potential to improve plant growth. Appl. Soil Ecol..

[26] Melo I.L.P.D., Freitas A.S.D., Barth O.M., Almeida-Muradian L.B.D. (2009). Correlation between nutritional composition and floral origin of dried bee pollen. Revista do Instituto Adolfo Lutz.

[27] Kullisaar T., Zilmer M., Mikelsaar M., Vihalemm T., Annuk H., Kairane C., Kilk A. (2002). Two antioxidative lactobacilli strains as promising probiotics. Int. J. Food Microbiol..

[28] Sárraga C., Carreras I., Regueiro J.A.G. (2002). Influence of meat quality and NaCl percentage on glutathione peroxidase activity and values for acid-reactive substances of raw and dry-cured *Longissimus dorsi*. Meat Sci..

[29] Jin G., He L., Yu X., Zhang J., Ma M. (2013). Antioxidant enzyme activities are affected by salt content and temperature and influence muscle lipid oxidation during dry-salted bacon processing. Food Chem..

[30] Naik S.R., Panda V.S. (2007). Antioxidant and hepatoprotective effects of *Ginkgo biloba* phytosomes in carbon tetrachloride-induced liver injury in rodents. Liver Int..

[31] Cheng N., Ren N., Gao H., Lei X., Zheng J., Cao W. (2013). Antioxidant and hepatoprotective effects of *Schisandra chinensis* pollen extract on CCl_4_-induced acute liver damage in mice. Food Chem. Toxicol..

[32] Li S., Pu X.P. (2011). Neuroprotective effect of kaempferol against a 1-methyl-4-phenyl-1,2,3,6-tetrahydropyridine-induced mouse model of Parkinson’s disease. Biol. Pharm. Bull..

[33] Šarić A., Balog T., Sobočanec S., Kušić B., Šverko V., Rusak G., Likić S., Bubalo D., Pinto B., Reali D., Marotti T. (2009). Antioxidant effects of flavonoid from Croatian *Cystus incanus* L. rich bee pollen. Food Chem. Toxicol..

[34] Coskun O., Kanter M., Korkmaz A., Oter S. (2005). Quercetin, a flavonoid antioxidant, prevents and protects streptozotocin-induced oxidative stress and β-cell damage in rat pancreas. Pharmacol. Res..

[35] Molina M.F., Sanchez-Reus I., Iglesias I., Benedi J. (2003). Quercetin, a flavonoid antioxidant, prevents and protects against ethanol-induced oxidative stress in mouse liver. Biol. Pharm. Bull..

[36] Hu C.H., Wang D.G., Pan H.Y., Zheng W.B., Zuo A.Y., Liu J.X. (2012). Effects of broccoli stem and leaf meal on broiler performance, skin pigmentation, antioxidant function, and meat quality. Poult. Sci..

[37] Reiter R.J., Tan D.X., Osuna C., Gitto E. (2000). Actions of melatonin in the reduction of oxidative stress. J. Biomed. Sci..

[38] Bartosz G. (2005). Superoxide dismutases and catalase. Reactions, Processes.

[39] Lee M.A., Choi J.H., Choi Y.S., Han D.J., Kim H.Y., Shim S.Y., Chung H.K., Kim C.J. (2010). The antioxidative properties of mustard leaf (*Brassica juncea*) kimchi extracts on refrigerated raw ground pork meat against lipid oxidation. Meat Sci..

[40] Hassan O., Fan L.S. (2005). The anti-oxidation potential of polyphenol extract from cocoa leaves on mechanically deboned chicken meat (MDCM). LWT-Food Sci. Technol..

[41] Lupea A.X., Pop M., Cacig S. (2008). Structure-radical scavenging activity relationships of flavonoids from *Ziziphus* and *Hydrangea* extracts. Rev. Chim..

[42] Silva M.M., Santos M.R., Caroço G., Rocha R., Justino G., Mira L. (2002). Structure-antioxidant activity relationships of flavonoids: A re-examination. Free Radic. Res..

[43] Hidalgo M., Sánchez-Moreno C., de Pascual-Teresa S. (2010). Flavonoid-flavonoid interaction and its effect on their antioxidant activity. Food Chem..

[44] Yang L.C., Li R., Tan J., Jiang Z.T. (2013). Polyphenolics composition of the leaves of *Zanthoxylum bungeanum* Maxim. grown in Hebei, China, and their radical scavenging activities. J. Agric. Food Chem..

[45] Zhishen J., Mengcheng T., Jianming W. (1999). The determination of flavonoid contents in mulberry and their scavenging effects on superoxide radicals. Food Chem..

[46] Li J., Wang F., Li S., Peng Z. (2015). Effects of pepper (*Zanthoxylum bungeanum* Maxim.) leaf extract on the antioxidant enzyme activities of salted silver carp (*Hypophthalmichthys molitrix*) during processing. J. Funct. Foods.

[47] Lawrence R.A., Burk R.F. (1976). Glutathione peroxidase activity in selenium-deficient rat liver. Biochem. Biophys. Res. Commun..

[48] Cakmak I., Marschner H. (1992). Magnesium deficiency and high light intensity enhance activities of superoxide dismutase, ascorbate peroxidase, and glutathione reductase in bean leaves. Plant Physiol..

[49] Marklund S., Marklund G. (1974). Involvement of the superoxide anion radical in the autoxidation of pyrogallol and a convenient assay for superoxide dismutase. Eur. J. Biochem..

[50] Shantha N.C., Decker E.A. (1994). Rapid, sensitive, iron-based spectrophotometric methods for determination of peroxide values of food lipids. J. AOAC Int..

[51] Salih A.M., Smith D.M., Price J.F., Dawson L.E. (1987). Modified extraction 2-thiobarbituric acid method for measuring lipid oxidation in poultry. Poult. Sci..

